# Editorial: Molecular pathogenesis and control of viral infectious diseases in children

**DOI:** 10.3389/fcimb.2024.1368324

**Published:** 2024-02-02

**Authors:** Jiali Cao, Chenguang Shen, Wanting He

**Affiliations:** ^1^ Department of Laboratory Medicine, Fujian Key Clinical Specialty of Laboratory Medicine, Women and Children’s Hospital, School of Medicine, Xiamen University, Xiamen, China; ^2^ BSL-3 Laboratory (Guangdong), Guangdong Provincial Key Laboratory of Tropical Disease Research, School of Public Health, Guangzhou, China; ^3^ Department of Laboratory Medicine, Zhujiang Hospital, Southern Medical University, Guangzhou, China; ^4^ Department of Immunology and Microbiology, The Scripps Research Institute, La Jolla, CA, United States

**Keywords:** viral infectious, children, pneumonia, diarrhea, epidemiology

Pneumonia and diarrhoea are the leading infectious causes of childhood morbidity and mortality. According to the WHO, pneumonia killed 740 180 children under the age of 5 in 2019, accounting for 14% of all deaths of children under 5 years old but 22% of all deaths in children aged 1 to 5 years. Approximately 1.6 million deaths occur each year globally due to diarrhea and diarrhea contributed to 15% of all under-five deaths (Black et al., 2010; Collaborators G B D D D, 2018). This Research Topic focuses on viral infections leading to pneumonia and diarrhea, featuring contributions on severe pneumonia pathogens, the impact of COVID-19 on RSV prevalence, climate factors influencing influenza virus circulation, and the treatment of rotavirus-induced diarrhea.

Pneumonia, stemming from various infectious agents, necessitates accurate diagnosis, particularly in severe cases. Li et al. utilized metagenomic next-generation sequencing (mNGS) alongside conventional tests for pathogen detection in samples from Pediatric Intensive Care Unit (PICU) patients. The study underscores the importance of advanced diagnostic techniques, such as mNGS, for a deeper understanding of severe pneumonia’s microbial epidemiology in pediatric patients. mNGS can identify unknown or novel pathogens in a single test, Not limited to pre-assumed pathogens. The detection rate of mNGS is much higher than that of conventional methods, especially for the detection of bacteria, fungi and RNA viruses, but the false positive results of mNGS detection are still a problem that cannot be ignored. Although the detection results of mNGS can provide richer information than the traditional detection methods, the etiological diagnosis still needs to be combined with conventional methods, and professional judgement and treatment by clinicians.

The emergence of COVID-19 in recent years has significantly influenced the trends of other respiratory pathogens. Li et al. observed a marked decrease in Influenza virus and adenovirus cases among PICU pneumonia patients during the COVID-19 pandemic. Studies from different regions indicate that non-pharmaceutical interventions during the pandemic reduced the prevalence of various respiratory pathogens and experienced subsequent “upsurge” (Agca et al., 2021; Rodgers et al., 2021; Wan et al., 2021; Garg et al., 2022; Alaib et al., 2023; Yang et al., 2023; Zhang et al., 2023). Jiang et al. conducted an analysis of a prospective long-term cohort monitoring project in Suzhou, China, examining epidemiological and clinical data of 59,934 lower respiratory tract infection patients. The study investigates the resurgence of RSV infections after the initial wave of COVID-19, offering insights to guide preventive actions ahead of potential resurgences following the Omicron variant. Notably, the reduction in COVID-19-related measures led to a significant increase in RSV infections during the second year of the pandemic, emphasizing the need for vigilance.

Influenza remains a significant viral infection among children, with meteorological factors playing a crucial role in transmission. Chen et al. explored the impact of climate factors on influenza virus circulation in Guangzhou, China, along with the unique influence of COVID-19 and related control measures on two influenza epidemics.

Rotavirus, a common cause of diarrhea in infants and young children, disrupts the balance between the host system and gut microbiota. Xu et al. investigated the impact of zinc-containing combined treatment on the intestinal microbiome, revealing a reshaped flora structure and an increase in short-chain fatty acid-producing flora, potentially regulating the host immune response.

This Research Topic aims to enhance our understanding of viral infections in children and provide fresh perspectives for effective management. Throughout this Research Topic, we witness the dedication of scientists, doctors, and researchers to children’s health, actively contributing to the response to viral infectious disease challenges.

## Author contributions

JC: Funding acquisition, Writing – original draft. CS: Writing – review & editing. WH: Writing – review & editing.
